# Spatially resolved spectroscopic differentiation of hydrophilic and hydrophobic domains on individual insulin amyloid fibrils

**DOI:** 10.1038/srep33575

**Published:** 2016-09-21

**Authors:** Tanja Deckert-Gaudig, Dmitry Kurouski, Martin A. B. Hedegaard, Pushkar Singh, Igor K. Lednev, Volker Deckert

**Affiliations:** 1 Leibniz Institute of Photonic Technology (IPHT), Albert-Einstein-Str. 9, 07745 Jena, Germany; 2 Chemistry Department Northwestern University, 2145 Sheridan rd, Evanston, IL 60208, USA; 3 Department of Chemical Engineering, Biotechnology and Environmental Technology, University of Southern Denmark, Campusvej 55, 5230 Odense M, Denmark; 4 Institute for Physical Chemistry and Abbe School of Photonics, University of Jena, Helmholtzweg 4, 07743 Jena, Germany; 5 Department of Chemistry, University at Albany, State University of New York, 1400 Washington Avenue, Albany, New York 12222, United States

## Abstract

The formation of insoluble β-sheet-rich protein structures known as amyloid fibrils is associated with numerous neurodegenerative diseases, such as Alzheimer’s and Parkinson’s disease. A detailed understanding of the molecular structure of the fibril surface is of interest as the first contact with the physiological environment *in vivo* and plays a decisive role in biological activity and associated toxicity. Recent studies reveal that the inherent sensitivity and specificity of tip-enhanced Raman scattering (TERS) renders this technique a compelling method for fibril surface analysis at the single-particle level. Here, the reproducibility of TERS is demonstrated, indicating its relevance for detecting molecular variations. Consequently, individual fibrils are systematically investigated at nanometer spatial resolution. Spectral parameters were obtained by band-fitting, particularly focusing on the identification of the secondary structure via the amide III band and the differentiation of hydrophobic and hydrophilic domains on the surface. In addition multivariate data analysis, specifically the N-FINDR procedure, was employed to generate structure-specific maps. The ability of TERS to localize specific structural domains on fibril surfaces shows promise to the development of new fibril dissection strategies and can be generally applied to any (bio)chemical surface when structural variations at the nanometer level are of interest.

Protein misfolding often results in the formation of β-sheet-rich aggregates, known as amyloid fibrils. Many unsolved structural questions of great interest in biochemistry are associated with these protein aggregates^1^,^2^,^3^. The development of efficient drugs for the treatment or prevention of amyloid fibril-related diseases such as Alzheimer’s or Parkinson’s would certainly benefit from a detailed surface characterization of fibrillar species. Various microscopic techniques, such as scanning or transmission electron microscopy (SEM and TEM) as well as atomic force microscopy (AFM), are commonly used to study protein aggregates. Although these approaches can supply morphological information at the nanometer scale, little chemical information for the analyzed sample can be obtained. Vibrational circular dichroism (VCD), solid-state NMR, IR and Raman spectroscopies have recently become valuable tools for fibril characterization^1^,^4^,^5^,^6^ but are limited by the requirement for a large sample volume and high protein concentration (~10 mM). This way, averaged information of the bulk volume of a sample is obtained but individual fibrils cannot be measured.

Amyloid fibrils typically exhibit high morphological and structural heterogeneity, a phenomenon known as fibril polymorphism^7^. Insulin forms tape-like fibrils at pH below 2 and twisted aggregates at pH above this point^5^. Despite significant tertiary and secondary structural rearrangements during fibrillation of insulin the disulfide bridges remain intact and are not cleaved^8^. Using NMR coupled with hydrogen-deuterium exchange and deep UV resonance Raman spectroscopy, it was possible to demonstrate that insulin did not decompose. Moreover, the amino acid residues, that were not accessible to the solvent and consequently had to be located in the fibril core, could be determined. Thus, all other amino acid residues are then located on the fibril surface and are accessible to the solvent. It was observed that one half of these amino acids was hydrophilic and the other hydrophobic. However, no information about the spatial distribution of hydrophilic and hydrophobic domains on the surface of the individual insulin fibrils could be obtained^8^.

Coupling Raman spectroscopy with scanning probe microscopy provides an access to both topography and molecular structure characterization in a single experiment with (sub-)nanometer resolution^9^,^10^,^11^,^12^,^13^.

Therefore, this approach known as tip-enhanced Raman scattering (TERS) becomes extremely valuable for structural characterization of individual amyloid fibrils. TERS is a highly sensitive and specific tool in this regard and is most valuable for studies at the single-molecule level^14^,^15^,^16^,^17^. So far it has been assumed that the spatial resolution of TERS depends on size, shape, metal of the nanoparticle at the tip apex. If the size of the metallic tip would predominantly determine the lateral resolution - a (sub-) nanometer resolution would be out of reach. Recent ultra-high vacuum low temperature TERS results on tetraphenylporphyrins^13^ have convincingly demonstrated a spatial resolution of 0.5 nm that cannot be explained by the classical concepts. Several recent quantum mechanical approaches also revealed that the high spatial resolution does not solely correlate with the tip apex’ diameter. Important are direct “chemical” interactions between the front-most tip features and the sample and also the particular atomistic composition of the metal nanoparticle. Both effects confine the local response area drastically^18^,^19^,^20^.

Several TERS studies on insulin fibrils^21^,^22^,^23^,^24^, collagen fibrils^25^ and other amyloid fibrils^1^,^26^,^27^ have been published so far and particularly for insulin it was demonstrated that the protein secondary structure of fibril surfaces clearly differs from the core: conventional and UV resonance Raman spectroscopy^28^ show that the latter consists of a pure β-sheet^8^, whereas surface-sensitive TERS reveals that the former is also composed of α-helix and unordered protein secondary structures as well as their mixtures^21^. In this context we conservatively assume that the surface area extends to a maximum of 2–3 nm because of the quickly decaying fields^19^,^29^.

In our previous TERS studies of different insulin fibril types, spectra were collected for arbitrary areas to gain statistically averaged information regarding the secondary structure and amino acid abundance on fibril surfaces^21^.

The present work pursues a systematic characterization of pre-defined areas on insulin fibrils grown at pH 1.5. These fibrils feature an almost flat topology and are presumed to grow by side-by-side with the association of intertwined protofilaments^21^. Initially we demonstrate the reproducibility and stability of the AFM-based TERS setup as being fundamental for analysis at nanometer resolution. In all ensuing experiments, TER spectra were continuously recorded on consecutive equidistant points along the fibril main axes. For a detailed data evaluation, spectra are analyzed using a band-fitting procedure, such that even amino acids with non-characteristic bands can be identified, enabling the distinction of mainly hydrophilic and hydrophobic domains. A two-dimensional amino acid distribution is visualized by generating maps from the fitted data. Finally, we show that data assessment by multivariate data analysis (N-FINDR) yields similar results.

## Results and Discussion

Prior to the actual TERS experiments discussed we specifically addressed the aspect of the instrumental reliability. Details on this first experiment can be found in Supplementary Figs S1 and S2. The repeated detection of transitions from one spectral pattern to the other indicates the reliability of the experiment where tip contamination and thermal drift could be excluded.

In the following sections it will be demonstrated that several amino acids can be directly identified in the TERS spectra with one or two marker bands, assuming that no other bands interfere (e.g. tyr, asn/gln). Other peptide features like cys-cys are known to show several bands due the presence of conformers. In this case up to six bands can be detected, simultaneously. The classification of amino acids with several overlapping modes requires a correlation of additional bands (e.g lys, his, gly) to ensure the assignment. The band assignment in Table 1 agrees with that in ref. 23 and the literature cited therein and is further supported by additional references where necessary. Overlap of bands is a particular issue when it comes to the assignment of the secondary structure from amide III bands^30^. Since amide III bands are sensitive to the conformation^31^ an assignment of modes between 1230–1300 cm^−1^ to amide III should be possible by correlating them with amide I bands. Since the absence of amide I bands in plasmon-enhanced spectroscopy is common and known such a correlation can be difficult^32^,^33^. Utilizing the amide III band region alone for a conformation assignment is challenging because other modes can interfere^34^,^35^,^36^,^37^ and must be explicitly excluded.

### Systematic surface characterization of insulin fibrils

**Figure 1:**
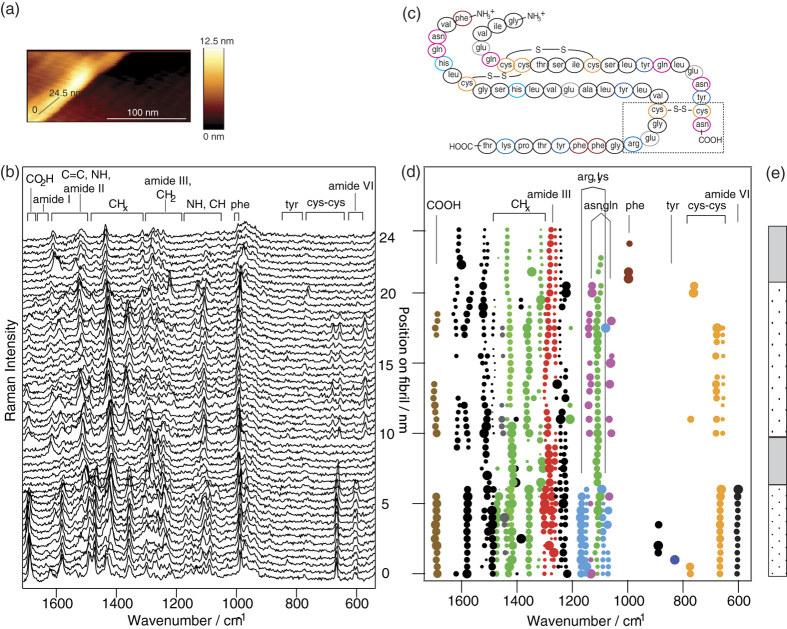
(**a**) AFM topography of an insulin fibril scanned during the TERS experiment. The gray 24.5-nm line indicates the investigated area. (**b**) Fifty raw spectra recorded along the fibril main axis with a step-size of 0.5 nm (λ = 532 nm, t_acq_ = 1 s); the three-letter codes refer to the respective amino acids. (**c**) Sequence of insulin (top: A-chain, bottom: B-chain) with the rectangle indicating the part of sequence that was most likely detected within the first 6 nm. (**d**) Selected fitted band parameters of the TERS spectra in (**b**) showing band intensity (spot) plotted versus the position on the fibril; the spot size corresponds to the band intensity and is normalized to each band separately; color code: cys-cys-orange; phe-dark red; asn, gln-magenta; amide III-red; arg, lys, N-terminal-light blue; CH, CH_2_-green; ala, val, ile, leu-gray; glu, C-terminal-brown; unspecific bands-black. (**e**) Schematic sketch of the surface hydrophilicity, domain with a mixture of hydrophilic and hydrophobic residues-dotted, domain with mainly hydrophobic character-gray.

In a second TERS experiment, information about structural organization was obtained along a 24.5-nm line on a fibril surface. In this case, 50 spectra along the fibril main axis (step-size: 0.5 nm) were collected, as indicated by the gray line in the AFM topography image in Fig. 1a.

The constant SNR of consecutively recorded raw spectra shown in Fig. 1b allow for straightforward data analysis. Due to similarities in functional groups, many amino acid residues cannot be immediately distinguished by their spectra. This applies, for example, to alanine, leucine, isoleucine and valine, which contain terminal CH_3_ groups. Nevertheless, heteroatom-containing functional groups or aromatic ring systems can clearly be instantly identified in the TER spectra since those bands do not overlap with other modes. For example, in Fig. 1b, several modes at 650–800 cm^−1^ (orange spots at 0–6.5 nm and again at 10–17.5 nm) could be assigned to cystine assemblies (cys-cys). Evidently, those modes appear at varying spectral positions and therefore must originate from different cys-cys residues with different molecular environments^23^,^38^,^39^,^40^. This is not surprising because the insulin sequence comprises six cysteine residues (pair-wise linked via a disulfide (S-S) bridge) with different conformations^8^. It must be noted that in all presented experiments, S-S modes were obscured by the silicon signal of the tip and consequently were excluded from further analysis. This also applies to C-C modes at approximately 950 cm^−1 ^35,38 overlapping with the silicon overtone (from the TERS tip). The consecutively detected bands at approximately 1700 cm^−1^ could be assigned to the carboxylic residues of glutamic acid (glu) and the C-termini^23^. The characteristic amino modes of asn/gln can be identified at 1060 cm^−1^ and 1135 cm^−1^ (see Table 1 and the complete dataset in Supplementary Table S3).

In the next step, all spectra were investigated using the previously mentioned band-fitting procedure. The obtained band intensities and spectral positions were plotted versus the local position on the fibril (see Fig. 1d). Bands detected within +/− 5 cm^−1^ were considered to be identical modes; such position variation always has to be accounted for and is a typical feature for small sample volumes, such as those used in TERS. Due to the high lateral resolution, only a few molecules are probed, and the statistical averaging of distinct band positions, such as in SERS, cannot be achieved^41^,^42^. It is apparent that several modes, represented by the light blue spots, were concurrently detected within the first 6 nm of the measured line. In comparison with previous SERS reports, those bands could be assigned to lysine (lys, 1070 cm^−1^, 1150 cm^−1^)^34^,^35^,^36^,^43^ and arginine (arg, 1090 cm^−1^, 1170 cm^−1^)^36^,^43^ and hence could be separated from asn/gln^23^ moieties. Coexisting bands in the νCH and νCH_2_ mode region (1470–1490 cm^−11^) most likely possess a high content of arg and lys modes (light blue in Fig. 1d). The simultaneous detection of arg, glu/COOH and cys-cys within the first 6 nm allows a tentative correlation of the spectral data and the position of the probed insulin sequence (rectangle in Fig. 1c). Bands at 1450 cm^−1^ appear only rarely and could be tentatively assigned to alanine (ala), valine (val), isoleucine (ile) and leucine (leu), according to previous SERS works^23^,^34^. All other CH and CH_2_ modes could not be assigned to specific amino acids and are marked green. The same is true for the black spots in Fig. 1d, with contributions from νC = C, ν CN, and νNH modes (1500–1620 cm^−1^) and νCH and amide III modes (1220–1250 cm^−1^). Identification of the discussed amino acids in Fig. 1c,d enables a differentiation of the investigated regions in domains with mainly hydrophobic character (gray) and domains with a mixture of hydrophilic and hydrophobic residues (dotted) on the fibril (see Fig. 1e). Within the first 6 nm a mixture of hydrophilic and hydrophobic residues was identified (dotted), as determined by the simultaneous detection of protonated amino acids (asn/gln, arg, lys) and non-polar cys-cys. Thereafter, the overall character of the domains alternates. The presence of such domains confirms recent AFM studies reporting the coexistence of hydrophobic and hydrophilic compartments on insulin fibrils via adhesion measurements^44^.

Finally, the secondary structure composition within the measured region will be addressed. Interestingly, the complete dataset lacks bands in the amide I region (1630–1679 cm^−1^, Table 1 and Fig. 1c,d), hampering direct secondary structure determination. This phenomenon is frequently observed in enhanced Raman spectroscopy, and its potential cause remains under discussion^32^,^33^,^38^. The amide III band region (1220–1300 cm^−1^) includes information on protein conformation as well, but an assignment is often difficult because CH_2_ modes of alkyl side chains^34^,^35^,^36^,^37^ can interfere. In the present experiment, the chemical composition of the surface varied within the measured 24.5 nm line, but bands at 1260–1300 cm^−1^ (red spots in Fig. 1d) were constantly present, with only slight position variations. This observation led to the assumption that those bands can be assigned to amide III modes (specifically α-helix/unordered structures^23^) rather than to side chain-specific modes. This assumption will be corroborated by the following experiments where amide I and amide III bands correlate and indicate α-helix/unordered structures. β-sheet structures are also known to be frequently present on pH 1.5 fibrils^21^, and the corresponding amide III bands are expected at 1230–1235 cm^−1^. Because bands in this spectral region in Fig. 1c,d vary strongly, a definite distinction from other modes was not possible. Considering our previous experiments on pH 1.5 fibrils it can be assumed that the spectra in Fig. 1b similarly contain a certain β-sheet structure contribution.

**Figure 2:**
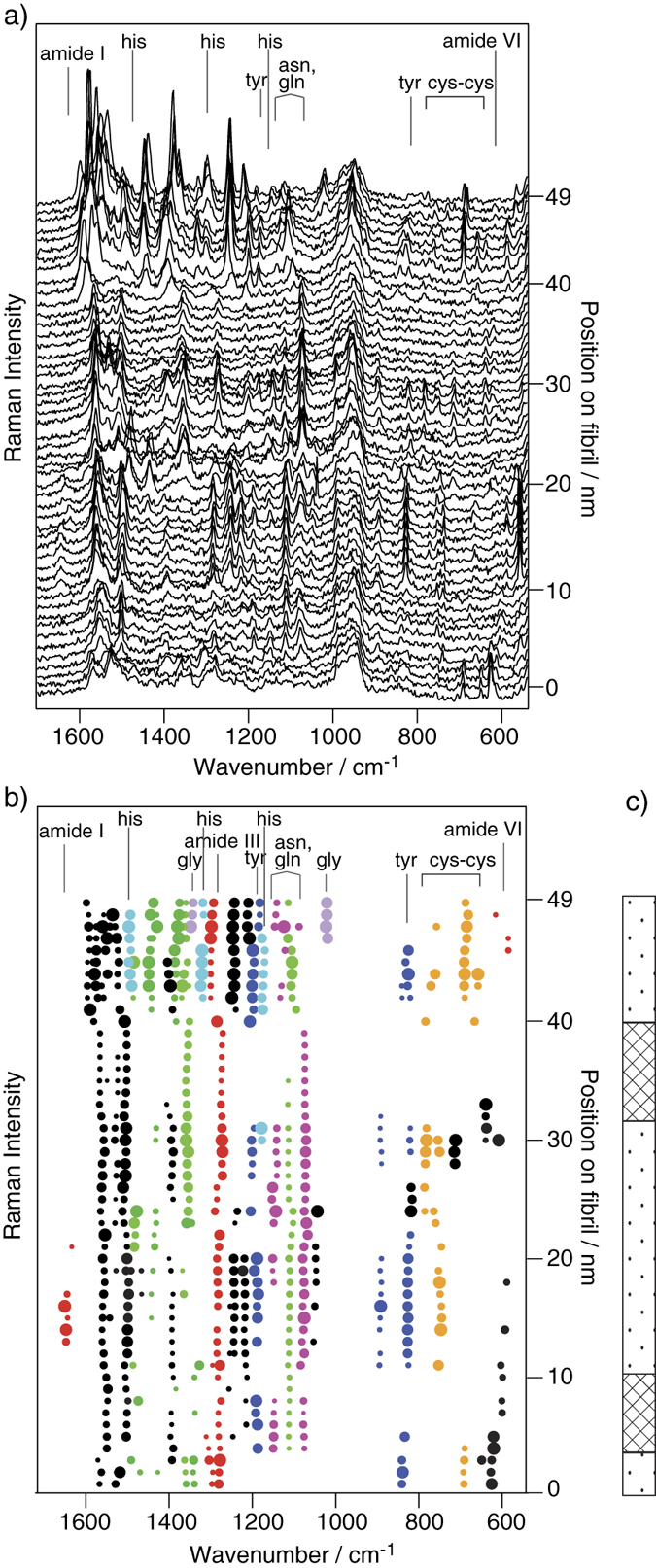
(**a**) Raw TERS spectra acquired along a 49.5-nm line (step-size: 1 nm) on an insulin fibril (λ = 532 nm, t_acq_ = 1 s). (**b**) Band fitting parameters plotted versus position on fibril; spot size corresponds to the band intensity and is normalized to each band; color code: cys-cys-orange; tyr-blue; asn, gln-magenta; amide I, III-red; his-turquoise; gly-lilac, CH, CH_2_-green; unspecific bands-black. (**c**) Schematic sketch of the surface hydrophilicity, mainly hydrophilic domain-checked; domains with a mixture of hydrophilic and hydrophobic residues-dotted.

In the third experiment investigating a different fibril with a different tip, the probed area was expanded to 49.5 nm (line with a point-to-point distance of 1 nm). Based on the first two experiments, it was concluded that although a larger step-size compromises the spatial resolution slightly, larger areas can be probed without the loss of spectral information. The acquired spectra (see Fig. 2a) were processed as above, and the respective band parameters plotted in Fig. 2b.

As in the previous experiments, cys-cys and asn/gln can be frequently found. This observation agrees well with the recently calculated propensity values for selected amino acids on insulin fibril surfaces^21^. The examined area in Fig. 1 is depleted of tyrosine, whereas Fig. 2 shows abundant tyr (blue spots in Fig. 2b). At first glance the repeated detection of this residue between ca. 10–20 nm of the measured line might give the impression of a much poorer spatial resolution compared to the fist two experiments. Due to the TERS tip preparation process it is only natural that not all tips show exactly the same resolution. With respect to the data in Fig. 1 and Supplementary Fig. S1 denoting a spatial resolution around 1 nm we assume that the resolution in Fig. 2 was within the same range. Most likely a cluster of tyr was present in this specific area of the fibril. Glycine (gly, lilac) and histidine (his, turquoise) can be identified at the end of the line: coexisting bands at 1020 cm^−1^ and 1320 cm^−1^ for glycine^34^,^45^ and three bands (1180 cm^−1^, 1330 cm^−1^, 1495 cm^−1^) for histidine (assignment see Table 1). It should be noted that a combination of bands was used for reliable amino acid assignment because a single band is not specific enough in most cases. The identification of polar and non-polar amino acids along the measured line in Fig. 2b enables again the classification of regions with mainly hydrophilic character (checked) and domains with a mixture of hydrohphilic and hydrophobic residues (dotted) in Fig. 2c. Similar to the previous experiment, domains with coexisting non-polar (cys-cys, gly) and polar (asn/gln, tyr, his) amino acids prevail in the measured region. Slight compositional differences compared to the previous fibril are not surprising and can be explained by the confined examined region and the often-reported polymorphism of insulin fibrils^21^,^22^.

Regarding secondary structure elements, amide I bands are only very rarely detected within a short range (14–18 nm of the measured line in Fig. 2). According to our previous work on homopeptides, all spectra of the gly containing peptide show amid I bands^32^. Consequently, one might expect amide I bands at the end of the measured line in Fig. 2a. However, in the case of insulin gly is embedded between amino acids with bulky side chains (e.g. phe). These bulky residues potentially lead to the suppression of amide I bands in our spectra. In contrast, bands in the amide III band region above 1260 cm^−1^ are ubiquitous, and the positions remain nearly unaffected despite changes in chemical composition. Consequently, as in the previous experiment, those bands could be assigned to α-helix/unordered structures; conversely, β-sheet structures in the amide III band region around 1230 cm^−1^ could not be definitively identified due to a likely overlap with the CH_2_ modes of cys-cys and tyr^34^,^35^,^36^,^37^,^38^. In previous studies, both residues were mainly found in β-sheet-dominated areas^21^,^22^, and it is very likely that the probed area, which is rich in cys-cys and tyr, has a large β-sheet contribution.

**Figure 3:**
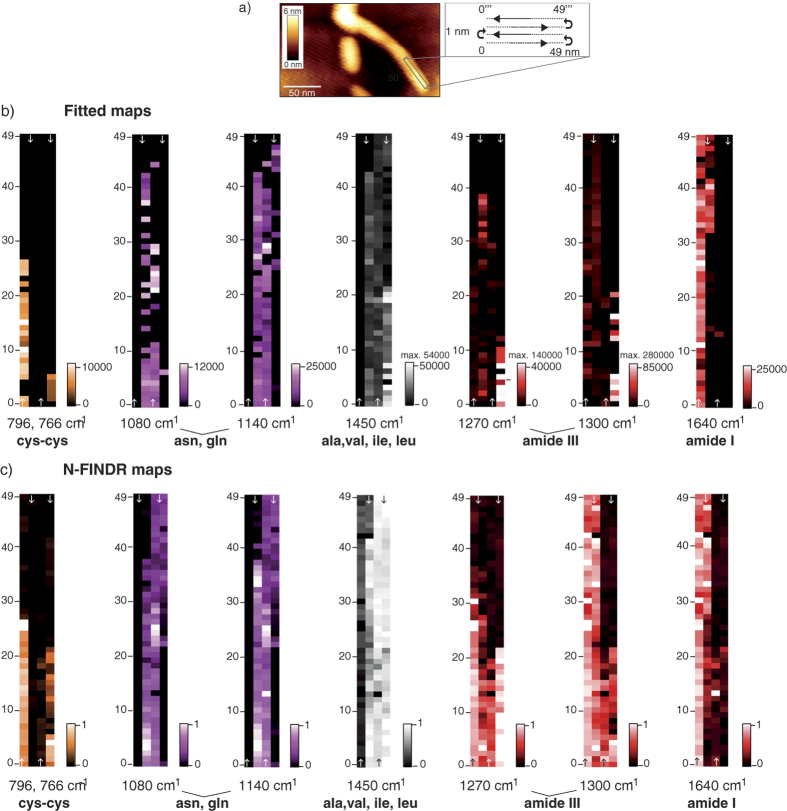
(**a**) AFM topography of a fibril for which 200 spectra on four 1 nm laterally shifted lines were recorded consecutively; each line: 49 nm with 50 equidistant measuring points; (λ = 532 nm, t_acq_ = 5 s. (**b**) Maps generated from the fitted band intensities; pixel brightness corresponds to band intensity. (**c**) Maps obtained from N-FINDR algorithm analysis; pixels represent the contribution of so-called “endmember” spectra (linear combination of spectra). All maps show the distribution of assigned amino acids and amide bands within the investigated area.

Finally, the same tip was used to measure along 4 parallel lines (lateral offset: 1 nm, each with 50 equidistant measuring points). In this way, the end of a fibril was probed, as indicated by the rectangle in the topography of Fig. 3a (all 200 raw spectra are available in the Supplementary Fig. S4; all bands are given in the Supplementary Table S3).

The complete dataset was analyzed as before. Additionally, the N-FINDR algorithm^46^, which has already successfully been applied to TERS spectra to distinguish cell membrane components (protein and lipids), was also implemented^47^. Regarding the graph in Fig. 3b, marker bands of selected amino acids and the peptide bond (amide bands) were fitted, and the obtained intensity values were plotted to obtain TERS maps of the fibril surface. In order to suppress outliers with very high signal intensities in the 1450 cm^−1^, 1270 cm^−1^ and 1300 cm^−1^ maps the color bars were set to lower intensity values than the maximum value. The maximum values are provided separately above the color bar. Sharp transitions denoting structural differences within each line and between lines are clearly perceptible. For instance, cys-cys was detected along the 1^st^ and 4^th^ lines, albeit at different positions (796 ad 766 cm^−1^, respectively, orange map). This observation again indicates cys-cys moieties with different chemical environments. In contrast, asn/gln (magenta maps) is absent on the 1^st^ line but almost continuously present along the 2^nd^ and 3^rd^ lines. Regarding the 4^th^ line, the tip was too far from asn/gln-rich regions. It should be mentioned that tyr was exclusively detected along the first line (see Supplementary Fig. S4) and was therefore omitted from mapping. Amino acids with methyl groups (ala, val, ile and leu, gray maps in Fig. 3b) are omnipresent on lines 2–4, with varying intensities. In comparison to the previous measurements, band fluctuations in the 1320–1470 cm^−1^ region are less prominent, and signals below 1300 cm^−1^ show an overall weaker enhancement along all four lines. Hence, it can be concluded that this specific fibril area has a widespread hydrophobic character with a more homogeneous surface composition compared to the previous species.

Information on the secondary structure is again provided by the position of amide I bands (see Table 1). Along the first two lines, α-helix/unordered structures obviously dominate, and bands in the amide III band region above 1260 cm^−1^ are present, even when amide I bands are not. This observation validates the general assignment of these bands to α-helix/unordered structures in all the presented experiments. The maps generated from the N-FINDR analysis^46^,^48^ (Fig. 3c) agree very nicely and provide comparable chemical information. Note that it is not possible to compare intensities of the maps in Fig. 3b,c since they were obtained from completely different data treatments. A considerable advantage of this method is that not all bands of the dataset have to be assigned, but the so-called endmember spectra, which are obtained from data analysis. Each endmember spectrum matches a raw spectrum at one specific point of the measured grid, whereas all other points of the dataset are a linear combination of all endmember spectra. In other words, N-FINDR considers the entire spectrum for the analysis, whereas in the band-fitting procedure, the user decides which bands are relevant. The endmember spectra and corresponding raw spectra for the present experiment are provided in Supplementary Fig. S5. The contribution of each endmember spectrum to the dataset can be imaged in terms of a map; adding up those images yields maps such as that shown in Fig. 3c, which illustrates the distribution of the selected amino acids and amide bands. Please see refs 46, 47, 48 for details regarding the N-FINDR method and its application on Raman and TERS spectra. Such multivariate analysis can be more straightforward and timesaving compared to the extensive assignment of single features necessary in the band-fitting procedure, particularly with large datasets. Nonetheless, an unequivocal advantage of the band-fitting procedure is the partitioning of bands and the visualization of correlating bands. In this way, non-specific modes can be assigned, possibly leading to the identification of further sample components. Clearly, the respective maps in Fig. 3b,c match nicely. Notable deviations in the amide III-fitted map and N-FINDR map are due to outliers (white pixels in Fig. 3b), which cause all other pixels to appear dark-red and are thus hardly distinguishable from amide III-free areas (black pixels). In particular, the sharp transitions in the orange cys-cys maps highlight the confined volume that is probed with the TERS tip and the high spatial resolution of the measurement.

## Conclusion

We present a systematic TERS investigation on spatially confined areas for four individual insulin fibrils. Clearly the experiments reveal the consistency of the acquired data and the stability of the setup as a basis for all of the presented data and the respective discussion. TER spectra, without any further enhancement of the so-called gap mode or any further molecular resonance enhancement, were collected at equidistant points along up to 50 nm-long lines on insulin fibrils with a point-to-point distance between 0.5 and 1 nm. This way the surface composition of the probed regions could be determined with extreme lateral resolution. Due to the intrinsically high resolution even amino acids without specific marker bands could be identified by exclusion arguments and spectra could consequently be assigned to an exact portion of the insulin sequence.

To the best of our knowledge, these experiments for the first time allow the identification and full spectral mapping of alternating hydrophobic, hydrophilic (or a mixture of both) nanometer-sized domains on insulin fibrils. These properties play the most significant role in amyloid fibril biological activity and are associated with toxicity as well as with the mechanism of fibrillation. It is noteworthy that this information is not readily accessible by any other technique. The subsequent data evaluation furthermore enabled the assignment of secondary structures based on amide III bands, which is important if the amide I spectral region provides insufficient information. Finally, to investigate the possibility of an automated method for analyzing large TERS datasets, spectra were assessed with the N-FINDR algorithm. The agreement of the maps generated from band fitting with those from N-FINDR analyses indicates that N-FINDR provides a convenient and automated method to obtain detailed information from a TERS dataset of a peptide. In particular, such a method is promising for a first assessment when considering much larger datasets.

The results are encouraging for future projects aimed at the identification and localization of interaction sites of amyloid fibrils with membranes and drugs. The presented combination of surface characterization and data evaluation is not restricted to protein assemblies but can be applied to a wide range of samples for (bio-)chemical surface analysis at the nanometer scale.

## Methods

### Sample Preparation

Bovine insulin (Sigma-Aldrich, St. Louis, MO) was dissolved (60 mg/mL) in HCl (pH 1.5) and heated at 70 °C for 2.5 hours without stirring. After cooling to room temperature, an aliquot of the fibrillar gel was re-suspended in HCl (pH 1.5) solution with a 1:100 dilution factor (V/V), and 10 μL was dropped onto a pre-cleaned cover slip. After incubation for 2–3 min, the suspension was gently sucked off from the glass surface with a pipette and washed twice with pH 1.5 HCl. The samples were dried under an argon flow prior to TERS measurements.

### TERS measurements

TERS measurements were performed using an AFM mounted on an inverted microscope, as previously described in detail in ref. 21. Commercial non-contact AFM cantilever tips (NSG10, NT-MDT) were evaporated with 25 nm silver and stored under argon until use. Closed loop feedback in the x and y directions was used to maintain the relative positioning uncertainty below 1 nm. The absolute drift of the system was detected by measuring a fibril sample and was between 15–40 nm/hour with a localization precision of 1–2 nm for fibril features measured on a 1 × 1 μm scan area. Experimental conditions were selected such that in these limits the maximum drift was 4–10 nm for the longest experiment. The nominal absolute positioning precision of the AFM’s closed loop system was 0.3 nm so the drift between two positions in an experiment is negligible ensuring a reliable basis for the relative location analysis. For excitation, a laser at 532 nm with a power of 770 μW was used on the sample. Depending on the activity of the tips, the acquisition time was set to 10 s (protofilament), 1 s (single lines) and 5 s (four lines) to achieve a signal-to-noise ratio allowing the assignment of at least 10 bands per spectrum. After each measurement, a reference spectrum was recorded for a substrate spot next to the fibril to exclude tip contamination^49^. All shown spectra are raw spectra and were neither baseline nor background corrected.

### Band-fitting procedure

Non-linear band fitting (Lorentzian profile) was accomplished using Igor Pro 6.12 (Wavemetrics, USA). Only bands with a signal-to-noise ratio above two were considered. Depending on the spectral areas of interest, up to ten bands were fitted simultaneously. For the graphs in Supplementary Fig. S2 and Figs 1 and 2, intensities were plotted as spots versus the measured position on the fibril. The spot size corresponds to the band intensity and is normalized to each band separately. The maps in Fig. 3b were generated from the fitted intensities of selected bands by plotting them versus a position of the fibril.

### N-FINDR analysis

Data were imported into MatLab v2013a (The Mathworks, Natick, MA) and analyzed with in-house written scripts^48^. The spectral region was limited to 600–1800 cm^−1^ and was vector normalized before being analyzed with the N-FINDR spectral unmixing algorithm using a non-negativity constrained fitting of endmembers^46^. A detailed protocol of the method can be found in ref. 48.

## Additional Information

**How to cite this article**: Deckert-Gaudig, T. *et al*. Spatially resolved spectroscopic differentiation of hydrophilic and hydrophobic domains on individual insulin amyloid fibrils. *Sci. Rep.* **6**, 33575; doi: 10.1038/srep33575 (2016).

## Supplementary Material

Supplementary Information

## Figures and Tables

**Table 1:** Marker bands in the TERS spectra of different insulin fibrils used for the identification of specific amino acids.

Experiment 1, tip 1, Figure S1	Experiment 2, tip 2, Figure 1	Experiment 3, tip 3, Figure 2	Experiment 4, tip 3, Figure 3	Assignment^23^
Wavenumber/cm^−1^				
660, 671–673, 686–688, 698	657–667, 680	658–666, 682–693		cys–cys (CS)
780, 793–806	760–775	750–770, 78–787	795–799	cys–cys (CS)
832–835, 845–848, 865–868	830	820–827, 834–840	819–826	tyr (ring breathing)
1060–1065, 1081–1083	1060–1067	1067–1078	1066–1074	asn, gln (NH_3_^+^)
	1070–1080			lys^34^,^35^,^36^,^43^ (NH_3_^+^, add. to 1150 cm^−1^)
	1088–1094		1083–1090	arg^36^,^43^ (=NH_2_^+^, add. to 1170 cm^−1^)
1121–1128	1130–1140	1122–1130, 1140–1150	1139–1148	asn, gln (NH_3_^+^)
	1145–1153			lys^34^,^35^,^36^,^43^ (NH_3_^+^, add. to 1075 cm^−1^)
	1166–1170			arg^35^,^36^ (=NH_2_^+^, add. to 1090 cm^−1^)
1210–1216	1206–1209	1186–1191, 1196–1204	1198–1192	CH, tyr^34^ (CH, add. to 825/855 cm^−1^)
1230, 1260–1274, 1284–1290, 1290–1299	1219–1229, 1230–1237, 1240–1249, 1251–1259, 1260–1267, 1271–1277, 1294–1305	1210–1225, 1240–1247, 1271–1276, 1293–1311	1237–1250, 1263–1270, 1271–1277, 1293–1308	amide III, CH_2_^34^,^35^,^36^,^37^
1638, 1644		1634–1648	1640–1650	amide I (α-helix/unordered)^31^
