# Supplementary material for: Spatially resolved spectroscopic differentiation of hydrophilic and hydrophobic domains on individual insulin amyloid fibrils
Source: Sci Rep. 2016 Sep 21;6:33575. doi: 10.1038/srep33575 (PMC5030623; doi:10.1038/srep33575)
Supplement: Supplementary Information [file srep33575-s1.doc]

**Spatially resolved spectroscopic differentiation of hydrophilic and hydrophobic domains on individual insulin amlyoid fibrils**

Tanja Deckert-Gaudig, Dmitry Kurouski, Martin A. B. Hedegaard, Pushkar Singh, Igor K. Lednev, Volker Deckert

**Instrumental reliability**

For this experiment protofilaments were used, which could be isolated when the insulin fibrillation process was stopped after 20 min according to ref. 1. Three parallel lines (lateral offset: 0.5 nm each) on an insulin protofilament (a direct precursor of a mature fibril) were probed step-wise.

The number of points per line was limited to 14 (step-size: 0.5 nm, total length: 6.5 nm), and spectra with a sufficient signal-to-noise ratio (SNR) of all bands were collected with an acquisition time of 10 s. In **S1** the raw spectra along the three lines are shown. A sharp transition after 2.5 nm from one pattern to another is apparent: tyrosine (tyr) bands (845 cm^-1^, 1215 cm^-1^, blue) disappear, while asparagine (asn) and glutamine (gln) bands (1060 cm^-1^, 1125 cm^-1^, magenta) appear. It is noteworthy that the structural similarity of asn and gln (side chains differ only by a CH_2_ group) makes a reliable differentiation of these amino acid residues impossible, with related modes potentially originating from either of the amino acids. Particularly the abrupt spectral transition at 2.5 nm in the 2^nd^ line of **S1** indicates the high spatial resolution of the experiment. To compare the data for all three lines in detail, the spectra were analyzed using band fitting procedures^2^. Band intensities of tyr and asn/gln marker bands are plotted as spots in **S2**.


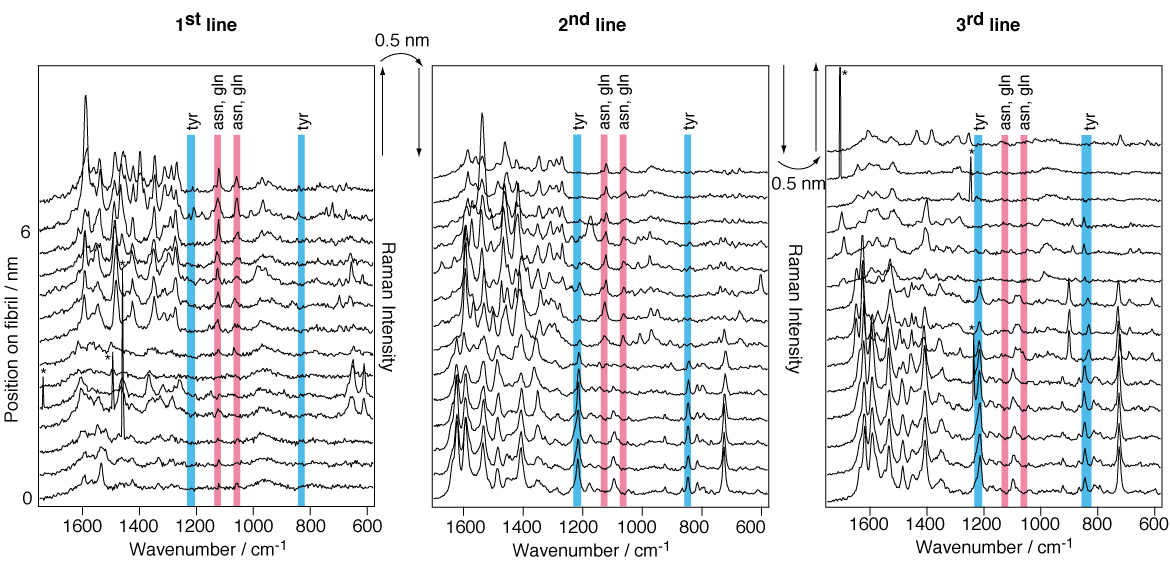


**S1.** Details of a TERS experiment in which spectra along three 6.5 nm-long lines (lateral offset 0.5 nm) were measured on an insulin protofilament (λ= 532 nm, t_acq_ = 10 s). Each line has 14 points (step-size 0.5 nm). Magenta lines indicate marker bands of asn/gln, and blue lines indicate marker bands of tyr, respectively. Cosmic rays are marked with an asterisk. All spectra are raw spectra.

.

| **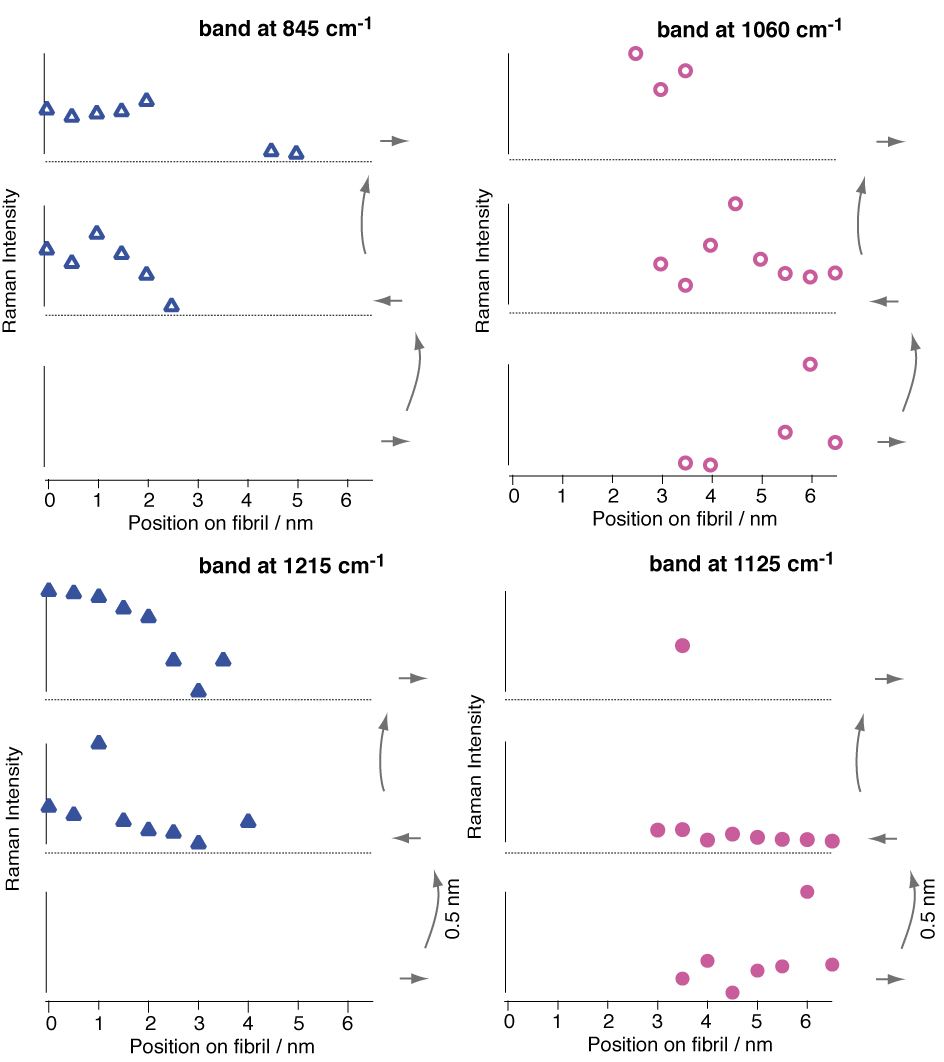** | **S2.** Band intensity plots of asn/gln marker bands detected along the three parallel lines of the spectra shown in S1; left: band intensity plots of tyr marker bands detected along the three parallel lines; right: band intensity plots of asn and gln marker bands detected along the three parallel lines, intensities were obtained from non-linear band fitting. Arrows indicate scanning directions. |
| --- | --- |

The plots of the marker bands of asn/gln, specifically the vibrational modes of the amino group (NH_3_^+^), demonstrate that these amino acids are repeatedly detected along the first and second lines. In contrast, tyr-related modes are repeatedly detected on the second and third lines. According to **S1** and **S2,** it is evident that bands at 845 cm^-1^ and 1210 cm^-1^ appear concurrently, enabling an assignment of the non-specific CH mode to tyr^3^. Notably, the presence of a single band at 830-840 cm^-1^ instead of a Fermi doublet (825/855 cm^-1^) is not unusual for tyr in proteins and is often observed if tyr is embedded in a hydrophobic environment (no hydrogen bonds)^4^. The spectral transition from one pattern to another within 2-3 consecutive steps points toward a spatial resolution of ~1 nm, which corroborates values determined from previous experiments on insulin fibrils^2,5^. The repeated detection of asn/gln and tyr on the three lines indicates the stability and reproducibility of the TERS setup. The observation of spectral changes in the nanometer regime validate that such high spatial resolution can be achieved with TERS under ambient conditions.

**Table S3. All bands and assignment of TERS spectra recorded on different insulin fibrils (S1 and Fig. 1-3 in the manuscript).**

| Experiment 1, tip 1,  Figure S1 | Experiment 2, tip 2,  Figure 1 | Experiment 3, tip 3,  Figure 2 | Experiment 4, tip 3,  Figure 3 | Assignment^2^ |
| --- | --- | --- | --- | --- |
| Wavenumber /cm^-1^ | | | | |
| 600, 611-617 | 601-606 | 589-616 | 602-609 | amide VI^6^ |
|  |  | 620-638 | 620-630 | amide IV^6^, tyr |
| 660, 671-673, 686-688, 698 | 657-667, 680 | 658-666, 682-693 |  | cys-cys (CS) |
| 720-728, 738 |  | 712-715 |  |  |
| 780, 793-806 | 760-775 | 750-770, 78-787 | 795-799 | cys-cys (CS) |
| 813 |  | 816-819 |  |  |
| 832-835, 845-848, 865-868 | 830 | 820-827, 834-840 | 819-826 | tyr (ring breath) |
| 890 | 890 | 890-895 |  |  |
| 900-905 |  |  |  | pro (ring) |
| 1008-1014 | 997 |  |  | phe (ring breath) |
| 1018-1020, 1030 |  | 1019-1023 |  | phe (CH), gly^3,7^ (CH, add. to 1320 cm^-1^) |
|  |  | 1044-1053 |  | CC^8,9^ |
| 1060-1065, 1081-1083 | 1060-1067 | 1067-1078 | 1066-1074 | asn, gln (NH_3_^+^) |
|  | 1070-1080 |  |  | lys^3,10-12^ (NH_3_^+^, add. to 1150 cm^-1^) |
|  | 1088-1094 |  | 1083-1090 | arg^10,12^ (=NH_2_^+^, add. to 1170 cm^-1^) |
| 1095-1098 | 1100-1110 | 1105-1115 |  | CC^8^ |
| 1121-1128 | 1130-1140 | 1122-1130, 1140-1150 | 1139-1148 | asn, gln (NH_3_^+^) |
|  | 1145-1153 |  |  | lys^3,10-12^ (NH_3_^+^, add. to 1075 cm^-1^) |
|  | 1166-1170 |  |  | arg^11,12^ (=NH_2_^+^, add. to 1090 cm^-1^) |
| 1172-1177 |  | 1170-1178 |  | his (NH, add. to 1330 cm^-1^ and 1495 cm^-1^) |
| 1210-1216 | 1206-1209 | 1186-1191, 1196-1204 | 1198-1192 | CH, tyr^3^ (CH, add. to 825/855 cm^-1^) |
| 1230, 1260-1274, 1284-1290, 1290-1299 | 1219-1229, 1230-1237, 1240-1249, 1251-1259, 1260-1267, 1271-1277, 1294-1305 | 1210-1225, 1240-1247, 1271-1276, 1293-1311 | 1237-1248, 1243-1250, 1263-1270,  1271-1277, 1293-1308 | amide III^13^, CH_2_^3,8,11,12^ |
| 1311-1317, 1323-1326 | 1310-1324 | 1319-1327 | 1319-1326 | CH_2_, gly^3,7^ (CH_2_, add. to 1020 cm^-1^) |
| 1347-1351, 1350-1355, 1364-1367, 1383-1386, 1396-1398, 1402-1407, 1410-1419, 1422-1427, 1434-1439, 1445-1449, 1453-1459, 1460-1465, 1483-1487 | 1340-1347, 1355-1368, 1385, 1400-1405, 1415-1419, 1420-, 1427, 1428-1438, 1450-1455, 1464-1475, 1480-1491 | 1341-1349, 1360-1370, 1375-1387, 1390-1406, 1427-1438, 1439-1446, 1467-1478 | 1328-1332, 1342-1349, 1350-1359, 1360-1365, 1370-1378, 1380-1385, 1390-1393, 1402-1406, 1418-1421, 1440-1446, 1447-1455 | CH, CH_2_, CH_3_, CN, his (CN, add. to 1175 cm^-1^ and 1495 cm^-1^) |
| 1499-1504 | 1504-1509 | 1490-1499, 1501-1505, 1507-1512 | 1492-1498, 1503-1510, 1511-1515 | his (NH, add to 1330 cm^-1^ and 1175 cm^-1^) |
| 1525-1535, 1540, 1560, 1588, | 1523-1529, 1578-1591 | 1523-1536, 1543-1557, 1560-1575, 1580 | 1523-1528, 1534-1552, 1560-1587 | amide II, NH, C=C |
| 1590, 1606 | 1607-1625 | 1590-1599 | 1592-1612 | phe, tyr (C=C), NH |
| 1638, 1644 |  | 1634-1648 | 1640-1650 | amide I (α-helix/unordered)^13^ |
|  | 1687-1700 |  |  | glu (COOH) |

**References**

1 Kurouski, D., Deckert-Gaudig, T., Deckert, V. & Lednev, I. K. Structural characterization of insulin fibril surfaces using tip enhanced Raman spectroscopy (TERS). *Biophys. J.* **104**, 49A-49A (2014).

2 Deckert-Gaudig, T., Kämmer, E. & Deckert, V. Tracking of nanoscale structural variations on a single amyloid fibril with tip-enhanced Raman scattering. *J. Biophoton.* **5**, 215-219 (2012).

3 Sjöberg, B., Foley, S., Cardey, B. & Enescu, M. An experimental and theoretical stud of the amino acid side chain Raman bands in proteins. *Spectrochim. Acta A* **128**, 300-311 (2014).

4 Thomas Jr., G. J. New structrual insights from Raman spectroscopy of proteins and their assemblies. *Biospectrosc.* **67**, 214-225 (2002).

5 Kurouski, D., Deckert-Gaudig, T., Deckert, V. & Lednev, I. Structure and composition of insulin fibril surfaces probed by TERS. *J. Am. Chem. Soc.* **134**, 13323-13329 (2012).

6 Miyazawa, T., Shimanouchi, T. & Mizushima, S.-I. Normal vibrations of N-methylacetamide. *J. Chem. Phys.* **29**, 611-616 (1958).

7 Xiaojuan, Y., Huaimin, G. & Jiwei, W. Surface-enhanced Raman spectrum of gly-gly adsorbed on the silver colloidal surface. *J. Mol. Struc.* **977**, 56-61 (2010).

8 Aguayo, T. *et al.* Raman and surface enhanced Raman scattering of a black dyed silk. *J. Raman Spectrosc.* **44**, 1238-1245 (2013).

9 Nsangou, M. DFT study of geometrical and vibrational features of small amino acids with polar side chains in hydrated media: L-threonine and L-serine. *Comp. Theor. Chem.* **966**, 364-474 (2011).

10 Garrido, C., Aguayo, T., Clavijo, E., Gómez-Jeria, J. S. & Campo-Vallette, M. M. The effect of the pH on the interaction of L-arginine with colloidal silver nanoparticles. A Raman and SERS study *J. Raman Spectrosc.* **44**, 1105-1110 (2013).

11 Zhu, G., Zhu, X., Fan, Q. & Wan, X. Raman spectra of amino acids and their aqueous solutions. *Spectrochim. Acta A* **78**, 1187-1195 (2011).

12 Aliaga, A. E. *et al.* SERS and theoretical studies of arginine. *Spectrochim. Acta A* **76**, 458-463 (2010).

13 Miura, T. & Thomas Jr., G. J. in *Subcell. Biochem.* Vol. 24 (eds B. B. Biswas & R. Siddhartha) 55-99 (Plemum Press, 1995).


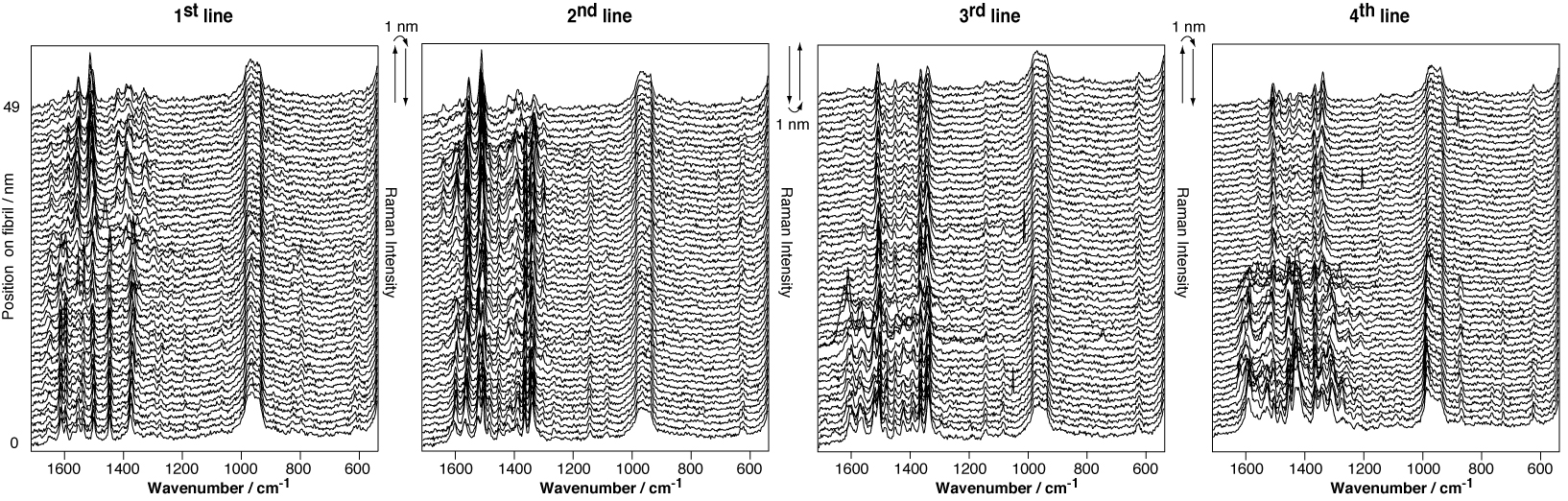


**S4.** Raw spectra of the experiment given in Figure 4 in the manuscript. The 200 raw spectra were recorded on four parallel lines (lateral offset 1 nm) on an insulin pH 1.5 fibril. Point-to-point distance along each line was 1 nm., t_acq_ = 5 s.


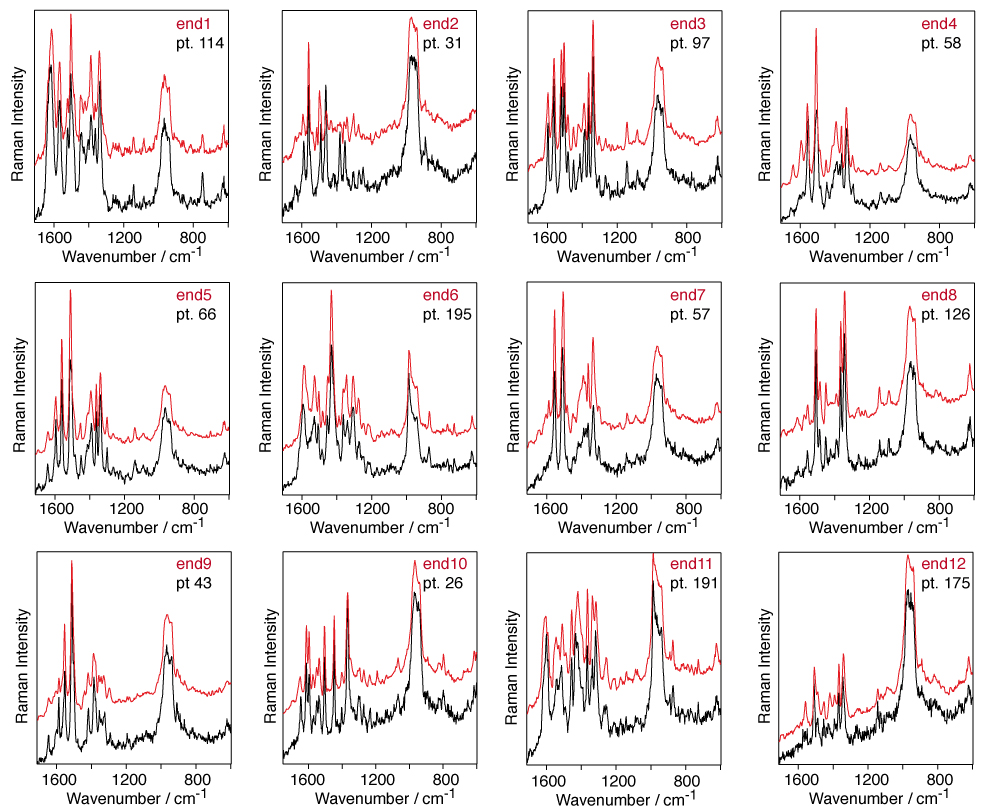


**S5.** Each graph shows an endmember spectrum (end, red) obtained from the N-FINDR analysis of the experiment given in SI3 and Figure 4c in the manuscript. The black spectra are the corresponding raw spectra of the experiment. Endmember spectra and raw spectra agree well, which is an indicator that the entire dataset can indeed be described by 12 endmember spectra and a linear combination of them.
